# Treatments of medical complications of anorexia nervosa and bulimia nervosa

**DOI:** 10.1186/s40337-015-0041-7

**Published:** 2015-04-05

**Authors:** Philip S Mehler, Mori J Krantz, Katherine V Sachs

**Affiliations:** Department of Medicine, University of Colorado Health Sciences Center, ACUTE at Denver Health, and Eating Recovery Center, 777 Bannock Street, MC4000, 80204, and 7351 E Lowry Blvd, Suite 200, Denver, CO 80230 USA; Department of Medicine, University of Colorado Health Sciences Center and ACUTE at Denver Health, 777 Bannock Street, MC4000, Denver, CO 80204 USA; Department of Cardiology, Denver Health and Department of Medicine, University of Colorado Health Sciences Center, 777 Bannock Street, MC4000, Denver, CO 80204 USA

**Keywords:** Treatment, Bulimia nervosa, Anorexia nervosa, Medical complications

## Abstract

Inherent to anorexia nervosa and bulimia nervosa are a plethora of medical complications which correlate with the severity of weight loss or the frequency and mode of purging. Yet, the encouraging fact is that most of these medical complications are treatable and reversible with definitive care and cessation of the eating-disordered behaviours. Herein, these treatments are described for both the medical complications of anorexia nervosa and those which are a result of bulimia nervosa.

## Background

As opposed to most other psychiatric disorders where there may be no medical complications associated with those illnesses, anorexia nervosa and bulimia nervosa inherently have many different medical complications. The specific treatments for those associated with anorexia nervosa and bulimia nervosa are described below.

### Anorexia nervosa

#### Treatment of secondary amenorrhoea and infertility

Secondary amenorrhea is a hallmark of anorexia nervosa. Although no longer considered a diagnostic criterion, it is nevertheless a nearly ubiquitous feature of severe weight loss and can often be a presenting feature of the disease [1,2]. The development of amenorrhea is most strongly correlated to loss of body weight. There is variability in the literature regarding the degree of weight restoration needed for resumption of menses, with some sources citing return to ninety percent of ideal body weight, and others seeing a stronger correlation with the weight at which cessation of menses was observed [3,4].

Although weight restoration is the mainstay of treatment for amenorrhea in the setting of anorexia nervosa, there has been some investigation into pharmacologic intervention targeted at the disrupted hypothalamic-gonadal axis. Leptin is a hormone that is secreted by adipocytes and functions as a mediator in the adaptation to energy deprivation. Women with hypothalamic amenorrhea have been shown to have low leptin levels compared to matched controls [5,6]. Two studies have demonstrated that administration of recombinant leptin can restore function of the hypothalamic-gonadal axis, with the resumption of menses [7,8]. However, these studies were performed in subjects, who despite having hypothalamic amenorrhea, had weights which were only in the low-normal range, and therefore the results have limited applicability to the overall anorexia nervosa population.

Therefore given the generally reliable resumption of menses with weight restoration, it would seem that there is generally little role for a pharmacologic intervention to achieve this effect sooner. Hence, it is, once again, inadvisable to use oral contraceptives in this setting singularly for the purpose of inducing a withdrawal bleeding for patients with anorexia nervosa. There has been essentially no clinical benefit demonstrated for estrogen replacement in this population [9]. Moreover, induction of withdrawal bleeding can give a false sense of wellness to these patients which potentially can temper motivation for ongoing necessary nutritional rehabilitation and weight restoration.

Despite lack of menses, it is possible for women with anorexia nervosa to become pregnant, and therefore it should not be assumed that contraception is unnecessary in these patients [10]. However, women with a history of anorexia are more likely to have future problems with fertility, and are more likely to have persistent amenorrhea regardless of weight restoration, as compared to the general population [11]. Women with a history of anorexia nervosa who do become pregnant are at greater risk for pregnancy complications such as hyperemesis gravidum, and spontaneous abortion, as well as adverse neonatal outcomes such as low birth weight [12,13]. Given the lack of clear correlation between features of anorexia nervosa and subsequent pregnancy complications, there is no clear treatment or preventative measure for this issue, other than treatment of the underlying eating disorder and nutritional deficiencies.

#### Treatment of bone disease

Decrease in bone mineral density is commonly found in anorexia nervosa. Therefore, it is estimated that patients with anorexia nervosa are three times more likely to have a fracture, compared with the general population [14]. The degree of bone loss has been correlated both with body mass index and duration of amenorrhea [15]. Contrary to the bone mineral density loss observed in post-menopausal women, bone loss in anorexia nervosa seems to be driven by a multifactorial process beyond just low estrogen, including hypercortisolemia, and reduced levels of androgens, leptin and insulin-like growth factor 1 (IGF-1). While the mainstay of treatment for bone density loss is weight gain and restoration of menstrual function, data have shown that decreased bone density may persist long after weight has again reached a normal level [16]. Based on this observation, efforts have been made to identify an adjuvant pharmacological therapy that could aid in bone density restoration, given its often early age of onset and long-term increased fracture risk.

Consideration has been given to the role of estrogen replacement therapy to target one of the many mechanisms leading to bone loss. However, there are little compelling data supporting this heretofore accepted practice. Additionally, as noted above, there exists concern that the use of estrogen in this setting, generally in the form of oral contraceptive pills, can in fact have deleterious effects by giving a false sense of normalcy [9]. There should actually be high reluctance to use it for this indication alone. Yet there is evidence of a lack of knowledge about this recommendation and many physicians continue to inappropriately prescribe estrogen therapy in anorexia nervosa [17]. While there exits one randomized controlled trial of 60 women with anorexia nervosa, that did show a statistically significant increase in bone density following treatment with oral contraceptive pills when coupled with recombinant human IGF-1 [18], further compelling data on this effect are lacking. In addition no clinical role has yet been adopted for IGF-1 in patients with anorexia nervosa.

Bisphosphonates have also been investigated as a possible treatment modality to increase bone density, given their successful use in post-menopausal women. However, due to the teratogenic nature of these drugs there is some concern about their use in women of childbearing age. It is known that bisphosphonates can remain in the body for many years after discontinuation, therefore raising concern for adverse effects on fetal development during pregnancies even years in the future. There are little data available about the outcomes of children born to mothers previously treated with these medications. Additionally, the data to support the efficacy of these medications in the anorexia nervosa population are somewhat limited. The most compelling study exploring this issue is a 2005 trial by Golden et al. in which 32 adolescents with anorexia nervosa were randomized to receive either alendronate or placebo. At one-year follow up, there was an increase in bone mineral density in both groups, correlated most closely with weight gain. The increase was greater in the alendronate group, but not significantly so compared with placebo [19]. There may be justification for their use in patients with severe osteoporosis and for males with normal testosterone levels. If the serum testosterone level is however low, then testosterone injection or patch therapy until the level returns to normal is reasonable [20].

Additional agents that have been considered as potential stimulants for bone formation and mineralization include, calcitonin, raloxifene, and testosterone, but none have been shown to have reliable effects in female patients with anorexia nervosa. There is one very recent study using teriparatide which showed substantial improvement in bone density after just six months of therapy in women with anorexia nervosa [21]. Calcium (1200 mg/day) and vitamin D (800 IU/day) are necessary for bone growth and development; however calcium supplementation is not sufficient by itself to prevent osteopenia in the setting of severe weight loss, nor is it effective treatment for decreased bone density once it exists [22].

Unlike most other medical complications of anorexia nervosa, bone density loss may not be fully reversible even with restoration to a normal weight. This is one of the few complications wherein the adverse effects may persist long-term, and arguably this may be one of greatest concern given their increased long-term fracture risk. Further studies to more fully explore the use of medications, validate the degree of associated risk, and investigate new agents will be necessary to provide more effective treatment.

Finally, some of the complications of anorexia nervosa do not require primary treatment once diagnosed beyond thoughtful reassurance for the patient and perhaps using the objective medical complication as leverage to try to exhort them to push on with nutritional rehabilitation and weight restoration. These include acrocyanosis, lanugo hair growth and the anemia, leukopenia and thrombocytopenia of anorexia nervosa. All of these resolve with successful treatment of anorexia nervosa.

#### Treatment of cardiovascular complications

The primary cardiovascular complications of eating disorders include hemodynamic, conduction system, and structural heart alterations. Among the most common alterations is orthostatic or relative hypotension even among ambulatory populations [23]. In general treatment of more significant hypotension can be accomplished with intravenous fluids among inpatients particularly if symptomatic, though hypotension improves with restoration of body mass. Hypotension may also accompany bradycardia making postural symptoms more pronounced.

Bradycardia is among the most common cardiac complications of anorexia nervosa, but absent high-grade atrioventricular block rarely requires cardiac subspecialist consultation. In rare cases, persistent junctional rhythm may be present in patients with severe anorexia nervosa. This may delay transitions of care from inpatient to outpatient treatment facilities and may lead to prolonged telemetry monitoring and increased hospital length of stay. In this case, we suggest provocative treadmill testing to demonstrate appropriate conversion to sinus rhythm in these patients as a means to assess cardiac electrical reserve [24]. Another common cardiac complication involving the electrical system is QTc interval prolongation, which has been postulated to underly the high risk of sudden cardiac death. However, even in patients with severe anorexia nervosa, marked QTc interval prolongation is uncommon when contributing factors such as hypokalemia are not present [25]. Expectant management of QTc prolongation is generally adequate including serial electrocardiography and telemetry monitoring in conjunction with electrolyte (potassium and magnesium) repletion and discontinuation of drugs known to prolong the QTc interval (including anti-emetics and antipsychotics). Patients with eating disorders also exhibit alterations in autonomic function and heart rate variability [26], though the clinical implications of these abnormalities are uncertain.

Another important issue in patients with restricting eating disorders is alteration in cardiac structure. Most commonly reductions in left ventricular mass are seen during starvation, but this is generally reversible. Some patients will develop pericardial effusions, which are generally self-limited and resolve with weight restoration. Pericardiocentesis is reserved solely for patients with evidence of cardiac tamponade by echocardiographic and/or clinical criteria. Recently, myocardial fibrosis (detected as late gadolinium enhancement on magnetic resonance imaging) was found in 23% of patients with anorexia nervosa [27]. This finding indicates the possibility of scar tissue and could theoretically underly a propensity to sudden cardiac death. Whether theses structural changes are reversible or etiologic with regard to arrhythmogenesis remain to be determined. Finally, mitral valve prolapse in association with the aforementioned left ventricular atrophy is very common due to changes in the valve annulus. However, even in the setting of mitral insufficiency, treatment remains expectant with serial echocardiography to document resolution. Overall, the cardiac complications of eating disorders although critical to recognize, are often reversible after supervised weight restoration.

#### Treatment of gastrointestinal complications

There are multiple gastrointestinal complications due to anorexia nervosa. Delayed gastric emptying is one of the most universal of these issues [28]. This condition is particularly problematic in that the symptoms it causes, postprandial fullness, bloating, and pain, further perpetuate the patient’s desire to avoid food. Definitive treatment is weight restoration which has been shown to improve both gastric emptying and associated symptoms [29]. Diet modification can serve as effective and sufficient treatment for some patients [30]. Dividing daily calories in to smaller meals eaten throughout the day, using liquid supplements and calorie-dense foods, avoidance of excessive fiber and perhaps small-particle food sized feeds can help mitigate some of the symptoms of gastroparesis.

However, for many patients symptoms are sufficiently problematic that they interfere significantly with weight restoration, and for these patients promotility agents can be considered. Metoclopramide, a dopamine receptor antagonist, has been found to be highly effective, even in small doses (2.5 mg), to hasten gastric emptying and reduce troubling symptoms of early satiety which interferes with nutritional rehabilitation. Appreciable symptom reduction can be seen with the first dose. Concern exists with the use of metoclopramide and the development of tardive dyskinesia. However, data are lacking regarding the risk of this effect specifically in the anorexia nervosa population, which tends to use very small doses of this medication (2.5 mg before meals) for limited periods of time. A meta-analysis suggests that the prevalence of this effect in the general population is in fact less than1%, a number that is significantly lower than the 1-10% risk quoted in national guidelines [31]. Other promotility agents sometimes considered for gastroparesis treatment include erythromycin and domperidone. However, data on these agents in this population are also lacking, and concerns regarding both safety and efficacy seem to relegate them to second choices after metoclopramide [32,33].

Superior mesenteric artery (SMA) syndrome is a rare etiology of abdominal pain and vomiting in patients with anorexia nervosa. It is caused by compression of the space between the superior mesenteric artery and aorta due to loss of the intervening mesenteric fat pad. In the majority of cases, profound weight loss is responsible for this anatomic phenomenon. Like gastroparesis, SMA syndrome is a serious complication of anorexia nervosa as the obstructive symptoms further exacerbate difficulties in achieving adequate caloric intake. The mainstay of treatment for SMA syndrome in anorexia nervosa is nutritional support, to restore the mesenteric fat pad and alleviate the obstruction. Enteral feeding, either with an oral liquid diet that can transverse the obstruction, or with a feeding tube placed into the jejunum, distal to the point of obstruction are effective short-term interventions [34]. There are case studies supporting the use of total parenteral nutrition (TPN) for short durations in cases of SMA syndrome refractory to enteral feeding, but the risks of parenteral nutrition must be very carefully considered in this vulnerable population [35]. The dysphagia and risk of aspiration seen in the more severely underweight patients with anorexia nervosa should be treated by well-informed speech therapists who are familiar with this issue in anorexia nervosa. Specialized diets, swallowing exercises and the temporary use of a stimulator applied to the throat area by these professionals, will, along with good nutrition, resolve their swallowing challenges [36].

Complaints of constipation frequently accompany weight loss in anorexia nervosa. This is a complex issue, as concerns regarding inadequacy of stool frequency and quantity are often highly influenced by the perception of what bowel habits the patient believes to be “normal” and also for desire perhaps from laxatives which the patient may believe reduce calorie absorption. However, due to decreased caloric intake and decreased motility of the gastrointestinal (GI) tract, slowed GI transit times and reflex hypofunction of the colon are real phenomena in anorexia nervosa [37]. With weight restoration colonic motility improves, but dietary modifications and medical therapy are often needed to treat and prevent progressive constipation and discomfort during that period of nutritional rehabilitation. Ensuring adequate fluid intake can often aid in symptom relief. Stimulant laxatives are not recommended, as not only can they also cause painful cramping but long-term, they can cause damage to colonic nerve cells. The preferred treatment modalities are osmotic laxatives, such as polyethylene glycol powder and lactulose, dosed based on the frequency of defecation [38].

Additional gastrointestinal problems that are seen in the context of anorexia nervosa and severe malnutrition include hepatitis and, rarely, pancreatitis. Markedly elevated transaminases, are often seen in the context of starvation. Case reports exist regarding recurrent idiopathic pancreatitis in eating disorder patients [39]. Both of these issues require supportive care in the acute phase. Rarely, elevation in liver function tests can be related to glucose and fat deposition during refeeding, which is termed steatosis, requiring a decrease in caloric advancement or a reduction in the amount of carbohydrate calories. An ultrasound can help distinguish between malnutrition induced elevations and those due to steatosis and can help avoid unnecessary slowing of refeeding – a modification that is only necessary in a small minority of cases and delays recovery if initiated inappropriately [40].

### Bulimia nervosa

#### Gastrointestinal

For patients with bulimia nervosa, treatment of their acid reflux symptoms follows the usual treatment plan generally used for any patient with this disorder, namely proton pump inhibitors, head of bed elevation and minimizing oral intake within a few hours of going to sleep. Although there are no data to suggest that patients with bulimia nervosa, who purge via self-induced vomiting, are at increased risk of esophageal carcinoma, good practice would be to refer a bulimic patient for upper endoscopy, if their acid reflux is refractory to standard therapy or anyone with dysphagia or a history of recurrent hematemesis; this is to exclude Barrett’s esophagus or even worse, neoplastic changes. Barrett’s remains a risk factor for esophageal carcinoma and endoscopic surveillance is recommended [41]. In addition, treatment of the dental consequences of self-induced vomiting largely revolves around ensuring good dental hygiene to reduce the harmful effects of stomach acid on teeth enamel. Brushing lightly with a fluoride-based toothpaste soon after vomiting is the current practice. Also, the sialadenosis which can often develop with abrupt cessation of self-induced vomiting, is treated with sialagogues such as tart candies, warm packs applied to the sides of the face multiple times per day and low dose anti-inflammatory agents such as ibuprofen. For refractory cases, a trial of prescription pilocarpine (sialogen) should be initiated before consideration of surgical treatments [42].

For those patients with bulimia nervosa who purge via stimulant laxative abuse, the treatment challenge which evolves is how to successfully “detox” them before they develop the cathartic colon syndrome, wherein their colon is relegated to an inert tube incapable of propagating fecal material resulting in severe constipation. There are no randomized controlled studies to inform how to optimally achieve cessation of stimulant laxative abuse while maintaining some degree of normal colonic function. Nor are there any data which speak to what amount of stimulant laxative usage is dangerous or over what duration of usage. Therefore a prudent approach seems to be one wherein the message delivered to these patients must be to completely abandon their usage versus simply reducing the number of stimulant laxatives used each day. Additionally, adequate fluid intake should be encouraged along with regular daily usage of an osmotic laxative, such as polyethylene glycol, to obligate intraluminal colonic absorption of water to assist with soft stools and successful defecation, even if that is not on a daily basis. Using the osmotic laxative, three to four times per day may be needed for those patients with a long history of daily stimulant laxative abuse.

Erythromycin in a dose of 250 mg two to three times per day, may be of utility to stimulate colonic transit, analogous to its usage in diabetic gastroparesis and other conditions where slow gastrointestinal transit occurs [43]. Medications such as Lubiprostone, which is approved for usage in patients with constipation-predominant irritable bowel syndrome, has never been rigorously studied in patients with bulimia nervosa who are attempting to discontinue their stimulant laxative abuse behaviors.

#### Fluid and electrolyte abnormalities

As mentioned in part two of this series, all modes of purging, which are engaged in excessively, will result in hypokalemia and metabolic alkalosis, although milder cases of diarrhea from laxative abuse, will cause a non-anion gap acidosis [44]. The mainstays of treatment for the hypokalemic metabolic alkalosis due to vomiting and diuretic abuse are intravenous saline and potassium. However, the efficacy of potassium repletion will be abrogated unless there is concomitant attention to correcting the hypovolemia via saline and reducing aldosterone secretion. Otherwise, urinary losses of potassium continue in response to elevated aldosterone levels. The key message to be aware of with the usage of intravenous saline is that it must be given at a slower than the typical rate used for dehydrated patients because these patients are in a strongly salt-avid state and can quickly put on 5–10 kilograms of edema fluid over the course of just a few hours; a result that is especially distressing to a patient population which is consumed by body image concerns. A prudent approach may be to admit such a patient to 24 hours observation and to run the intravenous saline at only 50-75 cc/hour for 24–36 hours [45]. Once the serum bicarbonate level normalizes into a range less than 30mmole/ml, it is safe to discontinue the intravenous saline. There are published guidelines as to when oral potassium repletion suffices and when the intravenous route is preferred [46]. In general for every 1 meq that the serum potassium level is less than 4.5 meq/L, the patient is estimated to have lost 100 meq of potassium. Thus, a critically low value of 2.5 meq/ml would suggest that there is a deficit of about 200 meq of potassium, assuming normal kidney function.

Importantly, for those patients with bulimia nervosa, who decide to abruptly cease their purging behaviors of either self-induced vomiting or laxative and diuretic abuse, pseudo-Bartter’s syndrome-induced edema formation can result in development of marked edema and exuberant weight gain over just a few days. Expectant proactive education must be offered that this risk exists so that these patients do not quickly revert back to their purging behaviors in order to mitigate against rapid and progressive weight gain. In addition, experienced clinicians typically prescribe spironolactone, once daily, in a dosage of 25 mg up to a rarely needed maximum dose of 200 mg, during the two to three weeks, after abrupt cessation of excessive and chronic purging behaviors, in order to block aldosterone and negate the propensity towards distressing edema formation and excessive weight gain [47].

Heretofore, low sodium diets have been incorporated into the treatment program for bulimia nervosa patients with this aforementioned propensity toward edema formation. This is similar to congestive heart failure patients where this measure has also been recommended despite a clear evidence base for its therapeutic effect. Recently, this has begun to be challenged as having no beneficial effect on weight loss or chemical stability in acute decompensated heart failure patients [48]. Presumably, the potential lack of efficacy may be due to salt restriction causing increased aldosterone secretion and perpetuating the underlying propensity towards edema formation that was being attended to with the usage of spironolactone. Indeed, there is emerging evidence that dietary salt restriction may activate mineralocorticoid receptor signalling and thus frustrate the efforts to successfully “detox” these patients with bulimia nervosa [49]. Thus, it may be prudent to relax the typical two gram sodium restriction-diet in this population of patients who are proceeding through the early stages of the absence of historical purging behaviors.

Readers may note that much of this section on fluid and electrolytes is also relevant to anorexia nervosa purging type and purging disorder.

## Conclusions

There are effective treatments for the medical complications of both anorexia nervosa and bulimia nervosa. With early recognition and appropriate medical treatment, patient with anorexia nervosa and bulimia nervosa may achieve successful treatment outcomes.

## Abbreviations

GI: Gastrointestinal
IGF-1: Insulin-like growth factor
SMA: Superior mesenteric artery
TPN: Total parenteral nutrition
